# Constant Magnetic Field as a Tool for Modification of the Properties of Polymer Composites with Silicone Rubber Matrix

**DOI:** 10.3390/ijms242316625

**Published:** 2023-11-22

**Authors:** Ewa Miękoś, Marek Zieliński, Michał Cichomski, Tomasz Klepka, Dorota Czarnecka-Komorowska, Dariusz Sroczyński, Anna Fenyk

**Affiliations:** 1 Department of Inorganic and Analytical Chemistry, Faculty of Chemistry, University of Lodz, Tamka 12, 91-403 Lodz, Poland; marek.zielinski@chemia.uni.lodz.pl (M.Z.); dariusz.sroczynski@chemia.uni.lodz.pl (D.S.); anna.fenyk@chemia.uni.lodz.pl (A.F.); 2 Department of Materials Technology and Chemistry, Faculty of Chemistry, University of Lodz, Pomorska 163, 90-236 Lodz, Poland; michal.cichomski@chemia.uni.lodz.pl; 3 Department of Technology and Polymer Processing, Faculty of Mechanical Engineering, Lublin University of Technology, Nadbystrzycka 36, 20-618 Lublin, Poland; t.klepka@pollub.pl; 4 Department of Plastics Division, Institute of Materials Technology, Poznan University of Technology, Piotrowo 3, 61-136 Poznan, Poland; dorota.czarnecka-komorowska@put.poznan.pl

**Keywords:** polymers, composites, constant magnetic field (CMF), silicone rubber, birch bark, expended graphite

## Abstract

The aim of this research was to obtain new polymer composites with a silicone rubber matrix, having favorable mechanical and functional properties. They contained admixtures in the amount of 10% by weight of expanded graphite (EG) or birch bark (BB). Additionally, some composites contained magnetic particles in the form of carbonyl iron in the amount of 20% by weight. The tensile strength, water absorption, frost resistance, surface contact angle, and free surface energy were examined. Microscopic images were taken using the SEM method and the content of some elements in selected microareas was determined using the EDXS method. In the study, a constant magnetic field with magnetic induction B was used, by means of which the properties and structure of polymer composites were modified. Scientific research in the field of polymers is the driving force behind the progress of civilization. Smart materials are able to respond to external stimuli, such as magnetic fields, with significant changes in their properties. The magnetic field affects not only chemical reactions, but also the crystallographic structure and physicochemical properties of the final products. Owing to their unique properties, such materials can be used in the space industry, automotive industry, or electrical engineering.

## 1. Introduction

The aim of this research is to use various types of admixtures and to influence the polymerization process with a constant magnetic field, thus expanding the spectrum of possibilities for designing materials with new, modified, and improved physicochemical properties. Polymer composites are commonly used, among others, in the chemical, construction and electrical industries, and therefore, an increasing demand for such materials has been observed for many years. Thus, developing new materials with similar or better properties than those currently produced has become extremely important. The applied constant magnetic field with magnetic induction B was an additional external factor that modified both the properties and structure of polymer composites. The effect of a constant magnetic field on the polymerization processes of polymer composites is relatively little known and rarely described in the literature.

Miękoś et al. [1] have investigated the impact of a constant magnetic field on changes in the physical and chemical properties of polymer composites based on polylactide (PLA) and epoxy resin. The examined composites contained admixtures in the form of magnetite (Fe_3_O_4_) and crystalline cellulose in the amount of 10%, 20%, and 30% *w*/*w* as well as starch in the amount of 10% *w*/*w*. Moreover, cellulose and starch fillers were added to provide partial biodegradability to the composites. Scanning electron microscopy linked with EDS and XRD techniques was used to characterize the chemical composition and structure of polymer composites, respectively. It was found that the polymer composites obtained in a constant magnetic field with a different magnetic induction B displayed changes of such properties as tensile strength, resistance to bending and impacts, water absorbability, frost resistance, and chemical resistance to acids and alkali. Thus, a magnetic field can be used as an external factor that allows materials with designed properties to be obtained, and in addition, natural additives accelerate the biodegradation processes of the composites.

Miękoś et al. [2] and Fu et al. [3] have also investigated the effect of a constant magnetic field on polymer composites with an epoxy resin matrix and natural fillers. They used birch bark, which contains botulin with bactericidal and virucidal activity, and yellow dextrin in the amounts of 20% *w*/*w*. As admixtures, expanded graphite and powdered graphite (20% *w*/*w*) were also used. During polymerization, the composites were placed between the poles of an electromagnet. In this case, under a constant magnetic field with magnetic induction B = 0.5 T, changes in some properties were detected. In particular, the hardness of the composite with an admixture of birch bark increased approx. 12% from 24.01 to 26.96 N/mm^2^ and with an admixture of yellow dextrin, it increased approx. 15% from 26.12 to 29.93 N/mm^2^. Moreover, the water absorbability of the pristine resin decreased from 0.18 to 0.13%, and that of the composite with powdered graphite decreased from 0.48 to 0.46%. Such changes in properties, often beneficial, are of great importance from the point of view of the potential use of these materials.

Since silicone rubber has excellent mechanical properties and high thermal stability, it is widely used in many branches of industry, including the aviation and mechanical industries. For example, silicone rubber is used for the production of sealants operating at high temperatures. However, with the development of industrial technologies, the difference between the thermal stability of the matrix and industrial requirements is gradually increasing. Therefore, the latest advances in innovation aim to increase the thermal stability of silicone rubber. Metal oxides such as CeO_2_, Al_2_O_3_, and Fe_2_O_3_, and carbon nanomaterials, e.g., carbon black, carbon nanotubes, and graphene, are widely used as additives, which increase the high-temperature resistance of silicone rubber composites [4].

Due to their numerous advantages, especially high hydrophobicity, composite materials based on silicone rubber gradually replace conventional porcelain and glass insulators, as written by Ullah et al. [5,6]. However, polymer-based materials underwent degradation when exposed to various external environmental factors, which resulted in a deterioration of their properties and a shortening of their lifespan. The aging characteristics of vulcanized silicone rubber HTV-SR filled with nanosilica and microcorundum at high temperatures were also investigated. Samples of materials with different compositions were subjected to variable environmental conditions in the aging chamber to simulate specific weather conditions during a 5000-hour-long experiment. The degradation of the composites was analyzed using FTIR spectroscopy, a static tensile strength test, thermogravimetry, and surface hydrophobicity measurements. The results showed that composites with a low content of nanosilica and microcorundum featured higher anti-aging properties compared to those with a high content of fillers of 2 and 10% *w*/*w*, respectively. The composite containing 2% *w*/*w* nanosilica demonstrated the highest anti-aging resistance.

Liu et al. [7] have investigated composites based on a transposable and highly thermally conductive silicone rubber with boron nitride as an admixture. These composites were prepared by hot pressing of layers at 180 °C. The composites were characterized by SEM, XRD, 2D WAXS, the finite element analysis method, and infrared thermography. It was found that the prepared composites feature a high thermal conductivity and a low interfacial thermal resistance [8,9,10,11,12].

Nanocomposites with magnetite and reduced graphene oxide have been obtained by their thermal decomposition and incorporation into a silicone rubber matrix [13]. The introduction of fillers followed by pressing and curing of the subsequent layers resulted in flexible composite elements with magnetic and heterogeneous features. The synergistic effect between dielectric losses and magnetic losses resulted in a high microwave absorption efficiency. The composites also showed excellent thermal conductivity, which allowed a rapid dissipation of the heat generated by absorbing microwave energy. Due to these properties, the obtained composites can be used in the aviation and electronics industries as microwave absorption devices [14,15,16].

An effective strategy for modifying the polymer properties is the use of nanofillers with a large specific surface area. Kumar et al. [17] used carbon nanotubes (CNT) and TiO_2_ as an admixture of silicone rubber-based composites. These fillers significantly improved the mechanical properties of the obtained silicone rubber nanocomposites. In particular, the elastic compressive modulus of composites without fillers was equal to 2.18 MPa and it increased to 6.8, 3.95, and 2.44 MPa for composites with CNT, CNT/TiO_2_, and TiO_2_, respectively. Similarly, the tensile strength of composites without fillers was equal to 0.54 MPa and it also increased to 1.37, 1.33, and 0.61 MPa for composites with CNT, CNT/TiO_2_, and TiO_2_, respectively.

The use of various types of admixtures and external factors affecting the polymerization process, for example, a constant magnetic field, significantly expands the possibilities for designing materials with new, modified, and improved physical and chemical properties. Our previous research on the effect of a constant magnetic field on chemical media demonstrated that it can improve both various parameters of the processes and the media properties. Silicone rubber and its composites have excellent mechanical properties and strong thermal stability. That is why they are widely used in industrial applications, e.g., in the aerospace and mechanical industries, for the production of sealants working at high temperatures. Polymer composites based on silicone rubber have begun to gradually replace conventional porcelain and glass insulators. On the basis of our research on polymer composites, it was found that those with an admixture of birch bark or birch bark with carbonyl iron are characterized by the highest water absorbability. The best effect, when the water absorbability of the composite decreased five times, was observed in a constant magnetic field with magnetic induction B = 0.5 T for a composite with an admixture of expanded graphite EG 290. An almost two-fold improvement in frost resistance, i.e., the lowest weight loss of the sample, occurred in the composite containing an admixture of carbonyl iron, especially when a constant magnetic field was operated. In addition, in a constant magnetic field, the polymer composites achieved anisotropic properties. Namely, the tensile stress of the composite material was much larger when the direction of tensile force **F** was perpendicular to the direction of magnetic induction B applied during the polymerization of the composite. The constant magnetic field also caused an increase in the wetting angle of the polymer composites along with a decrease in the surface free energy, and consequently, the surface of composites became more hydrophobic. Moreover, the polymer composites prepared in a constant magnetic field had visible and large clusters of magnetic particles, which were arranged in parallel chains.

A constant magnetic field affects the structure of polymer composites. Such thermodynamic functions as enthalpy and internal energy change during the polymerization process. Thanks to magnetic particles, the process of changes in the physicochemical properties of polymer composites is controlled by means of a magnetic field. As a result, the absorbability of polymer composites decreases, and their chemical resistance to acids and alkalis, as well as frost resistance, increases.

## 2. Results and Discussion

### 2.1. Water Absorbability and Frost Resistance

**Figure 1:**
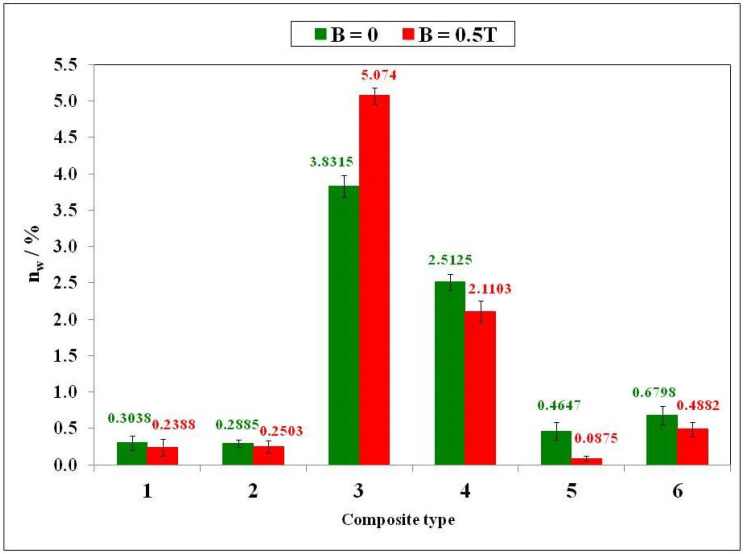
Comparison of water absorbability (n_w_) of polymer composites prepared in a constant magnetic field with magnetic induction B = 0.5 T and without a magnetic field.

Water absorbability defines the water absorption capacity of a given material, in this paper a polymer composite, and is a measure of its maximum water saturation. Figure 1 shows the water absorbability of the six tested polymer composites as a function of their composition prepared in a constant magnetic field with magnetic induction B = 0.5 T and without a constant magnetic field.

The polymer composites with an admixture of birch bark (sample 3) and birch bark with carbonyl iron (sample 4) are characterized by the highest water absorbability. In comparison with the reference sample 1 with silicone rubber matrix, the water absorbability increased 10- and 20-fold for composites prepared without and with a constant magnetic field, respectively. The highest effect was obtained for composite 5 with an admixture of expanded graphite EG 290 prepared in a constant magnetic field (B = 0.5 T) for which the water absorbability decreased five-fold from 0.4647 to 0.0875%.

**Figure 2:**
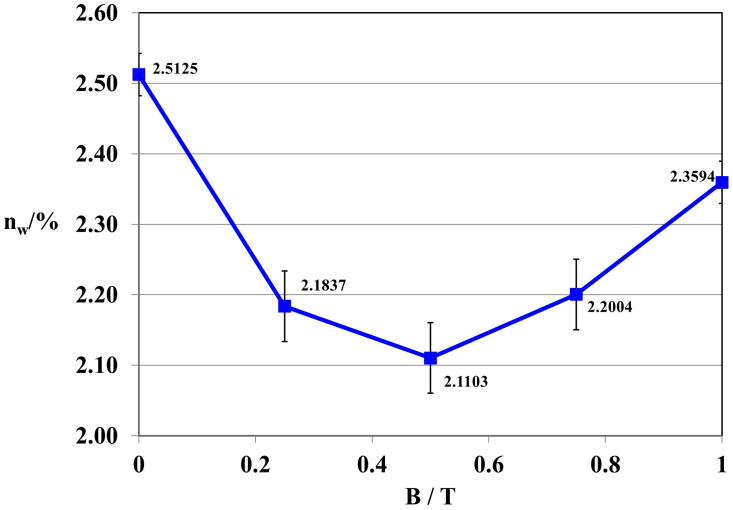
Water absorbability of polymer composite 4 GUM(B)OL-1(Fe)(BB) as a function of magnetic induction B.

**Figure 3:**
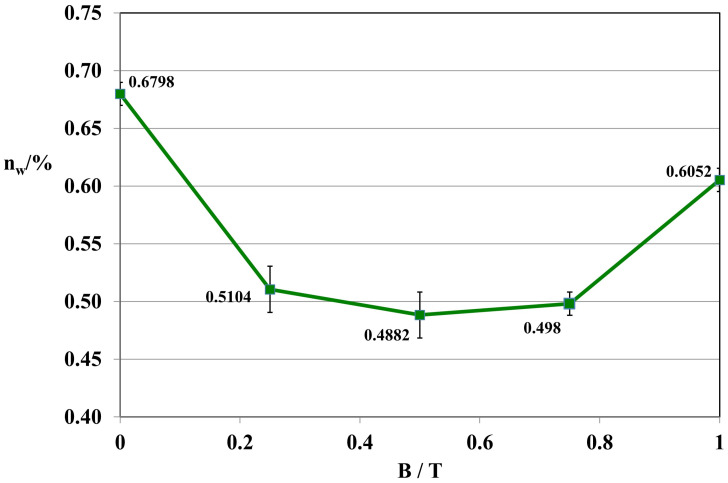
Water absorbability of polymer composite 6 GUM(B)OL-1(Fe)(EG) as a function of magnetic induction B.

Composites 4 [GUM(B)OL-1(Fe)(BB)] and 6 [GUM(B)OL-1(Fe)(EG)] (Table 5) were selected to present detailed studies of the influence of a constant magnetic field with a wide range of magnetic induction B varying from 0 to 1 T on the water absorbability of polymer composites. These dependencies are shown in Figure 2 and Figure 3.

The tests demonstrated that the magnetic induction of B = 0.5 T was the most effective in reducing the water absorbability of composite 4 GUM(B)OL-1(Fe)(BB) in a constant magnetic field (Figure 2). The same result was observed in the case of polymer composite 6 GUM(B)OL-1(Fe)(EG) (Figure 3).

**Figure 4:**
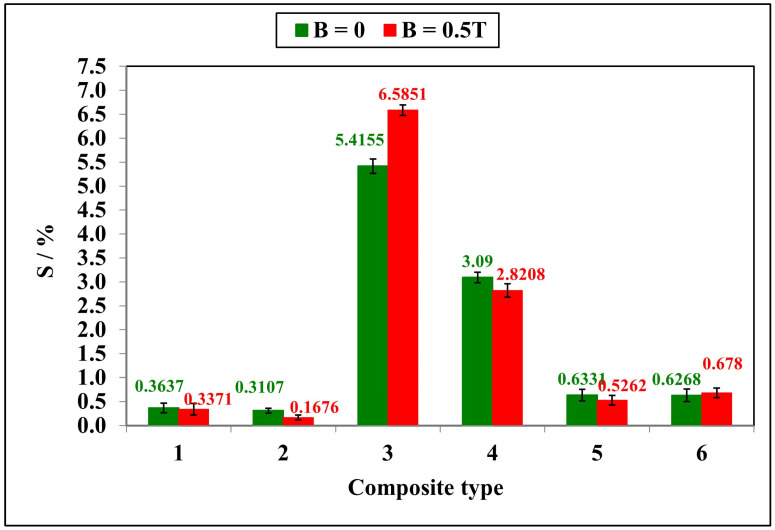
Frost resistance (S) of polymer composites prepared in a constant magnetic field with magnetic induction B = 0.5 T and those prepared without a magnetic field.

The results of the frost resistance tests of the six polymer composites are presented in Figure 4. The tests demonstrated that the exposure of polymer composites to a negative temperature caused an increase in the volume of water absorbed in the composites during the transition to a solid state. In addition, the cracking of samples was a negative effect of water crystallization during the freezing of composites.

The reference sample 1 GUM(B)OL-1(−)(−) containing silicone rubber showed a frost resistance of 0.3%, both when it was prepared in a constant magnetic field B = 0.5 T and without a magnetic field. The deterioration of frost resistance, i.e., a reduction in weight loss of the sample, was the most significant for sample 3 with birch bark as an admixture. The frost resistance decreased 18 and 22 times for the samples prepared without and with a constant magnetic field with induction B = 0.5 T, respectively. An improvement in frost resistance, i.e., almost a two-fold decrease in the weight loss of the sample, was found for sample 2 with carbonyl iron as an admixture prepared in a constant magnetic field.

**Figure 5:**
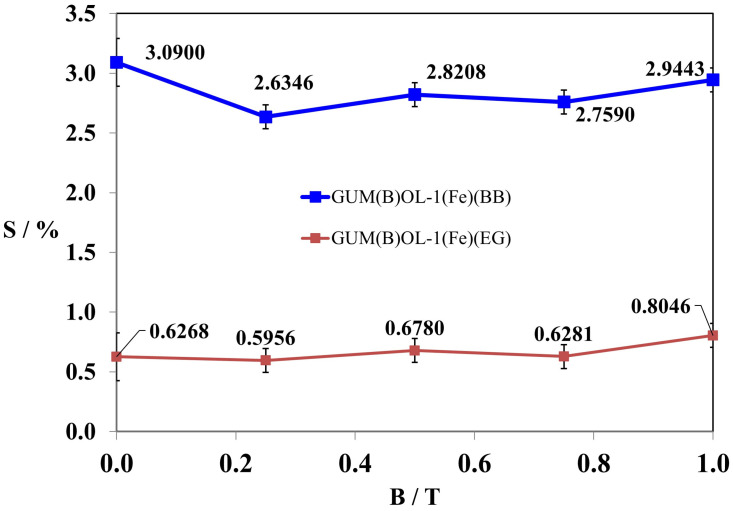
Frost resistance (S) of polymer composites 4 GUM(B)OL-1(Fe)(BB) and 6 GUM(B)OL-1(Fe)(EG) as a function of magnetic induction B.

In order to examine the impact of a constant magnetic field with a magnetic induction B in the range of 0–1 T on the frost resistance of polymer composites, the samples 4 GUM(B)OL-1(Fe)(BB) and 6 GUM(B)OL-1(Fe)(EG) were selected. The results of the studies are presented in Figure 5.

The obtained results show that in all the ranges of magnetic inductions used, the frost resistance of polymer composite 4 GUM(B)OL-1(Fe)(BB) with carbonyl iron and birch bark as an admixture is higher, i.e., the weight loss of the sample is lower, than that of polymer composite 6 GUM(B)OL-1(Fe)(EG) with carbonyl iron and expanded graphite as an admixture. In addition, the frost resistance of polymer composite 6 GUM(B)OL-1(Fe)(EG) does not change significantly up to a magnetic induction B of 0.8 T. However, when the magnetic induction B was equal to 1 T, the frost resistance of this polymer composite increased by about 20%.

### 2.2. Mechanical Properties

For the study of the influence of a constant magnetic field on tensile strength in the static tensile test of polymer composites, composite 6 GUM(B)OL-1(Fe)(EG) (Table 5) was selected.

**Figure 6:**
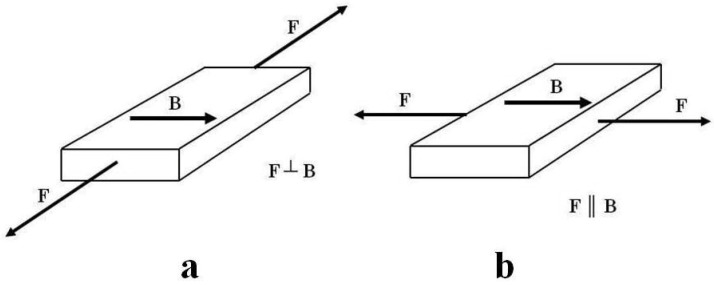
Method of mechanical tensile treatment of composites: (**a**) tensile forces F are directed perpendicular to the direction of action of the magnetic induction vector B; (**b**) tensile forces F are directed parallel to the direction of action of the magnetic induction vector B.

The composites were obtained in a constant magnetic field within the range of 0–1 T. In addition to determining how magnetic induction affects the mechanical strength and elongation of polymer composites, it has also been demonstrated that the composites achieved anisotropic properties in a constant magnetic field. Anisotropic properties depend on the direction of testing these features; hence, the composites were stretched in different directions. A sample with dimensions of 8 cm × 5 cm × 0.5 cm obtained in a constant magnetic field was used for the tests. The method of mechanical tensile treatment of the composites is shown in Figure 6.

**Figure 7:**
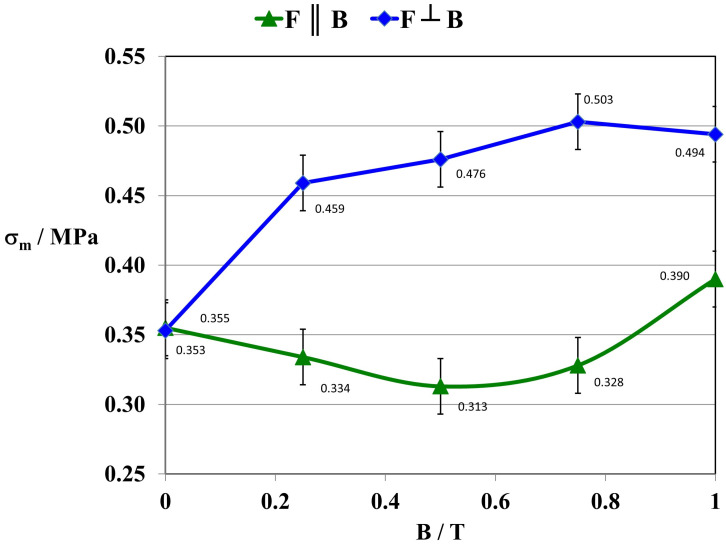
Maximum stress (σ_m_) of composite 6 GUM(B)OL-1(Fe)(EG) as a function of magnetic induction B.

By studying the **σ_m_** stress values in the composite material, it was found that they are much higher when the direction of tensile force **F** is perpendicular to the direction of magnetic induction B (Figure 7). Under a constant magnetic field, the magnetic particles of carbonyl iron were arranged in chains consistently with the direction of the magnetic induction B vector, providing additional strength reinforcement to the composite. Tearing apart such a sample required the use of a greater force. This is very important from the perspective of the potential use of the composite in practice. The influence of the direction of tensile forces F on the composite, when stretched, caused a difference of approx. 55% of the stress value in the composite material, exposed to a constant magnetic field B = 0.5 T during polymerization.

**Figure 8:**
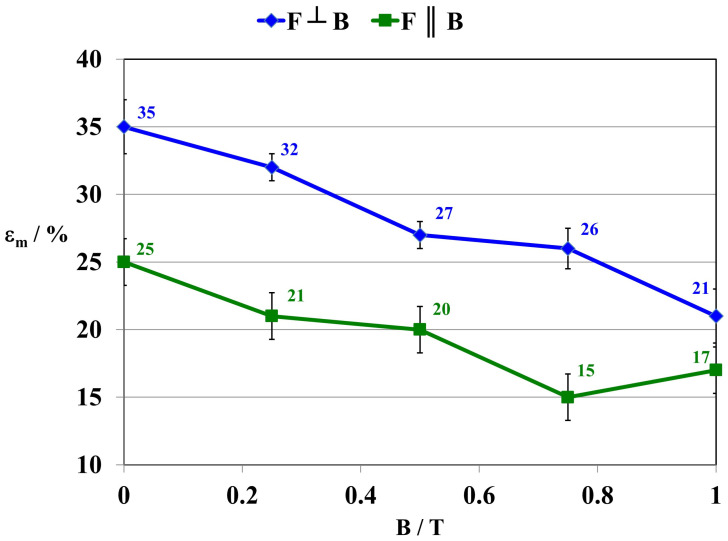
Dependence of deformation during tensile strength test (Ɛ_m_) on magnetic induction B for composite 6 GUM(B)OL-1(Fe)(EG).

The research also included an examination of the effect of a constant magnetic field on the deformation of the polymer composite during stretching (Ɛ_m_). In this case, the influence of the direction of tensile forces F relative to the direction of action of the magnetic induction B vector was determined (Figure 8).

Over the entire range of values of the tested constant magnetic field, the deformation values of the material decrease. Depending on the direction of action of tensile forces F on the composite, the difference in deformation of the material, e.g., for composites formed at B = 0.5 T, amounted to approx. 35%.

### 2.3. Surface Wetting Angle and Surface Free Energy

Surface wetting angle θ is the angle formed between the flat surface of a solid and the plane tangential to the surface of the liquid bordering that solid. In particular, the wetting angle of the surface by the liquid θ is a measure of the hydrophilic/hydrophobic properties of this surface; namely, if θ < 90°, then the wettability of the surface by the liquid is high (hydrophilic surface), and if θ > 90°, then the wettability of the surface by the liquid is low (hydrophobic surface).

**Figure 9:**
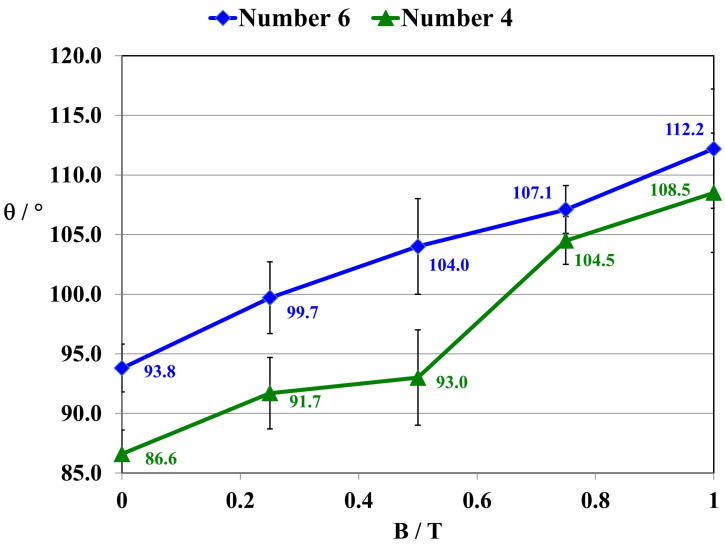
Surface wetting angle by water (θ) of polymer composites 4 GUM(B)OL-1(Fe)(BB) and 6 GUM(B)OL-1(Fe)(EG) with silicone rubber matrix, obtained in a constant magnetic field environment within the range of 0–1 T.

The determinations involved measurements of six measuring drops of the following liquids: water, diiodomethane, and glycerin. On the basis of these measurements, the values of the surface wetting angle and the surface free energy of the composites were determined. For the purpose of studies of the influence of a constant magnetic field on the surface wetting angle of polymer composites within the range of magnetic flux density values (B = 0.0–1.0 T), polymer composites 4 GUM(B)OL-1(Fe)(BB) and 6 GUM(B)OL-1(Fe)(EG) were selected. The results of the measurements of the wetting angle of the surface by water θ are shown in Figure 9.

On the basis of the conducted research, the constant magnetic field was found to cause an increase in the surface wetting angle of polymer composite 4 GUM(B)OL-1(Fe)(BB) with an admixture of carbonyl iron and birch bark by 25.3%, while for composite 6 GUM(B)OL-1(Fe)(EG) with the admixture of carbonyl iron and expanded graphite, the increase was 19.6%. The wettability of the surface of these composites was demonstrated to decrease due to the increase in the hydrophobicity of the samples.

**Figure 10:**
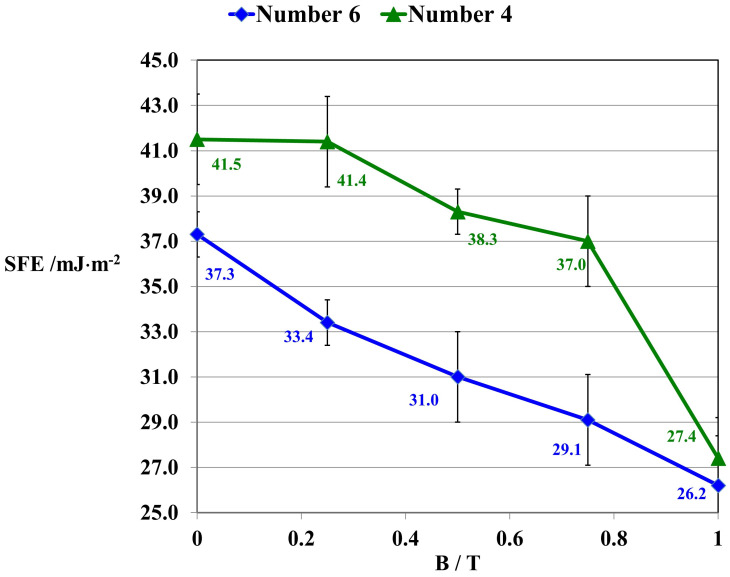
Surface free energy (SFE) of polymer composites 4 GUM(B)OL–1(Fe)(BB) and 6 GUM(B)OL–1(Fe)(EG) with silicone rubber matrix, obtained in a constant magnetic field environment within the range of 0–1 T.

Surface free energy (SFE) is one of the thermodynamic quantities describing the equilibrium state of atoms in the surface layer of a specific material. Surface free energy reflects a specific state of imbalance in intermolecular interactions present at the interface of the phases of two media. The surface free energy is equal to the work needed to form a unit of area during the separation of two equilibrium phases in a reversible isothermal process. To present studies of the influence of a constant magnetic field on the surface free energy of polymer composites within the range of magnetic induction (B = 0–1 T), composites 4 GUM(B)OL-1(Fe)(BB) and 6 GUM(B)OL-1(Fe)(EG) were also selected. The results of the calculations of surface free energy are shown in Figure 10.

As a result of the conducted research, the constant magnetic field was found to cause a decrease in the surface free energy of polymer composite 4 GUM(B)OL-1(Fe)(BB) of 34%, and 29.4% in the case of composite 6 GUM(B)OL-1(Fe)(EG). This was due to the fact that as a result of the action of the constant magnetic field, the surface of the material was less rough, the filler grains and polymer particles were more packed, and there was less free space in between. Ferromagnetic particles aligned themselves in the direction of the magnetic induction vector, so that other particles, even diamagnetic ones, were also arranged in a certain order [2].

### 2.4. Microscopic Evaluation of Surface Morphology of Polymer Composites

**Figure 11:**
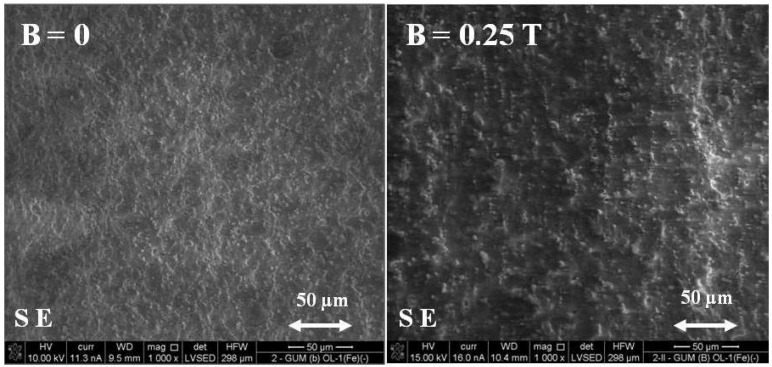
Secondary electron (SE) image of the GUM(B)OL-1(Fe)(−) composite, obtained without a magnetic field (B = 0) and with a constant magnetic field (B = 0.25 T).

The research was carried out using the FEI Quanta 3D FEGSEM scanning electron microscope with an additional FIB ion column, integrated into the EDAX Trident system. SEM images were recorded using images of backscattered electrons (BSE) and secondary electrons (SE) in low vacuum mode. Analysis of the chemical composition in microareas was performed by X-ray energy dispersion spectroscopy (EDXS—Apollo 40 company EDAX) using the ZAF correction procedure. The X-ray spectra (qualitative and quantitative microanalysis) were recorded. In the example of polymer composite 2, with an admixture of carbonyl iron obtained without exposure to a magnetic field (B = 0) and under a constant magnetic field (B = 0.25 T), differences in the morphology of the surface of these composites were demonstrated. The secondary electron (SE) image of composite 2 is shown in Figure 11.

**Figure 12:**
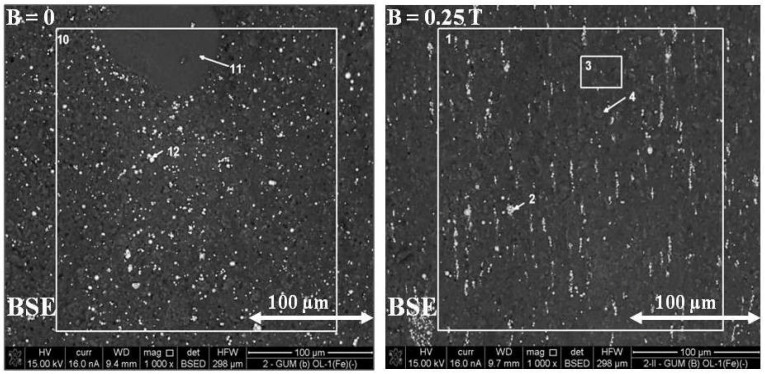
Backscattered electron image (BSE) of the GUM(B)OL-1(Fe)(−) composite, obtained without a magnetic field (B = 0) and with a constant magnetic field (B = 0.25 T).

Based on Figure 12, it can be seen that polymer composite 2 GUM(B)OL-1(Fe)(−), obtained in a constant magnetic field of B = 0.25 T, is characterized by larger clusters of magnetic particles, arranged in chains.

The backscattered electron image (BSE) of polymer composite 2 GUM(B)OL-1(Fe)(−) is shown in Figure 12.

**Figure 13:**
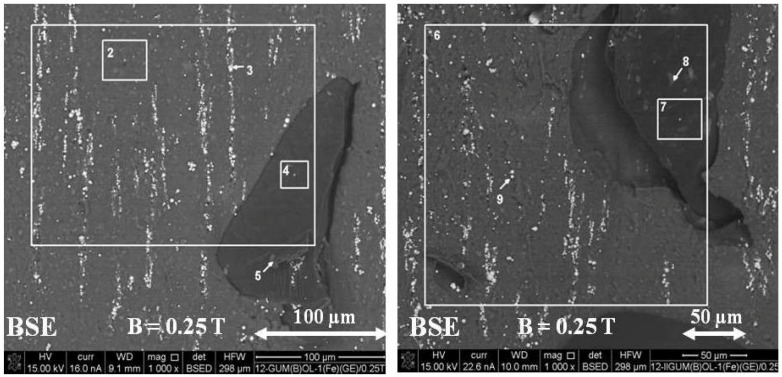
Backscattered electron image (BSE) for composite 6 [GUM(B)OL-1(Fe)(EG)] obtained with a constant magnetic field (B = 0.25 T).

The images were divided into microareas, which were numbered, and the chemical composition was analyzed using the EDXS method. The quantities of some components in the microareas are shown in Table 1. For composite 2, obtained in a constant magnetic field B = 0.25 T, chains of magnetic particles formed according to the direction of the magnetic induction vector B were visible. In microarea 10, silicon (Si) (53.9%) was predominant due to its contribution to silicone rubber, as well as iron (Fe) (11.8%), due to its magnetic particle content. Microarea 11 is also dominated by silicon (Si) (57.6%), and there are fewer iron particles (2.8%), as shown in Figure 13. Microarea 12 shows a definite predominance of carbonyl iron particles (73.2%).

Table 2 shows the microareas in which the content of the particular components in polymer composite 2, obtained in a constant magnetic field of B = 0.25 T, was determined (Figure 12). In area 1 (general), silicon (Si) (55.4%) dominates due to its contribution to silicone rubber. Area 2 shows carbonyl iron particles (64.4%). Areas 3 and 4 show again the predominance of the main component—silicone rubber, i.e., silicon (Si) (53.3% and 52.9%, respectively).

The analysis of composite 6 GUM(B)OL-1(Fe)(EG) (Table 5), obtained with the participation of a constant magnetic field (B = 0.25 T), showed that the presence of expanded graphite significantly affects the changes in the morphology of the material surface. For polymer composite 6, obtained in a constant magnetic field B = 0.25 T, the presence of chains of magnetic particles formed according to the direction of the magnetic induction vector B was found.

Table 3 shows microareas 1 to 8, in which the content of certain components in polymer composite 6, obtained in a constant magnetic field B = 0.25 T, was determined (Figure 13). Areas 1, 2, 5, 6, and 7 show the predominance of the main component, silicone rubber, i.e., silicon (Si) (52.5%, 52.6%, 48.4%, 60.1%, and 51.8%, respectively). Area 3 demonstrates the predominant content of carbonyl iron particles (69.4%). Since the quantitative result for carbon (C) is overestimated, as this component is derived from expanded graphite and silicone rubber, it was not included in quantitative studies. However, when analyzing areas 4 and 8, a lower silicon content (23.5% and 14.2%), indicating the presence of expanded graphite, can be observed.

### 2.5. Linking of Some Parameters Confirming Changes in Properties of Polymer Composites in a Constant Magnetic Field

To confirm the changes in the properties of polymer composites that occur during the exposure to a constant magnetic field, several parameters from different tests have been cited simultaneously, for the same samples (Table 4). These were the parameters for the selected polymer composites 4 [GUM(B)OL-1(Fe)(BB)] and 6 [GUM(B)OL-1(Fe)(EG)], characterizing various admixtures and having magnetic particles.

For composite 4 [GUM(B)OL-1(Fe)(BB)], there is a decrease in water absorbability (n_w_) in a constant magnetic field with magnetic induction B = 0.5 T from 2.51% to 2.11%. This is confirmed by the increase in the surface wetting angle of the composite (θ) from 86.6 to 93.0 (grade). As the angle (θ) increases, the wettability of the surface decreases and the surface becomes more hydrophobic. At the same time, the surface free energy (SFE) of the polymer composites is reduced from 41.5 to 38.3 mJ·m^−2^. For composite 6 [GUM(B)OL-1(Fe)(EG)], similar changes can be observed. The water absorbability (n_w_) in a constant magnetic field with magnetic induction B = 0.5 T decreases from 0.68% to 0.49%. This is confirmed by the increase in the surface wetting angle θ of the composite from 93.8 to 104.0°. This is also confirmed by mechanical strength tests. With a more favorable effect of the force **F** relative to the direction of magnetic induction B (**F**⊥**B**), the maximum stress in the composite (σ_m_) increases from 0.35 to 0.48 Pa. At the same time, the tensile deformation (Ɛ_m_) is reduced from 35.0 to 27.0%. Therefore, the effect of a constant magnetic field involved a reduction in the roughness of the material surface and more dense packing of the filler grains and polymer particles so that there was less free space between them.

The effect of a constant magnetic field on atoms manifests itself in the form of stresses in the crystal lattice. In liquids, however, magnetic fields acting on both electrons and ionized atoms cause dynamic effects, one of which is the volumetric motion of the medium. In turn, the movement of the masses causes the fields to be modified. Thus, we are dealing with a coupled system of matter and fields. During the polymerization of composites, an interaction between the liquid and solid phases takes place. Capillary interactions (attractive forces), Van der Waals interactions (attractive forces), electrostatic Coulomb interactions in the double layer (repulsive forces), expansion pressure (repulsive forces), and hydrodynamic interactions occur between grains. When the particles are small in size (10^−6^–10^−2^ mm) (colloidal particles), the electrostatic Coulomb forces and Van der Waals forces predominate, while when the particles are larger (0.1–1 mm), the capillary forces begin to prevail. Hydrodynamic forces become important only when a constant magnetic field is applied. The magnetic particles contained in the polymer, in order to achieve large magnetic moments, align themselves in the direction of the external magnetic field. The intermolecular interaction forces cause the particles to attract each other, which leads to their aggregation in complex networks, shortening the distance between them, and thus stiffening the material. The change in the internal energy U or enthalpy H in the magnetic field is due to the direction of charges in the material or ions in the liquid, as well as the disappearance of the insulating thermal barrier.

In the magnetic field, the absorbability of polymer composites decreases and their chemical resistance to acids and alkalis as well as frost resistance increases. Owing to their unique properties, these materials can be widely used in the space, electrical engineering, or automotive industries.

## 3. Materials and Methods

### 3.1. Components of Polymeric Composites

The following components were used for the preparation of polymer composites: GUMOSIL B silicone rubber (manufactured by the “Silikony Polskie” Sp. z o.o. Chemical Plant, Nowa Sarzyna, Chemików 1, Poland); OL-1 hardener (manufactured by the “Silikony Polskie” Sp. z o.o. Chemical Plant, Nowa Sarzyna, Chemików 1, Poland); EG 290 expanded graphite with high resistance to corrosion, elevated temperatures, electromagnetic radiation and compression [expanded graphite (EG) is also referred to as intumescent graphite. It is produced by the treatment of flake graphite with various intercalation reagents, such as acids. When flake graphite, after the intercalation process, is heated at high temperatures (approx. 1000 °C), the intercalation compounds break down into gaseous products that increase the pressure between the graphite layers. This high pressure creates enough force to push the graphite planes outwards. This results in an approx. 300-fold graphite volume increase, a huge increase in its surface area, as well as a reduction in the bulk density of graphite. Expanded graphite has several unique properties, such as high corrosion resistance, heat resistance, radiation resistance, compressive resistance]; dried birch bark, with a high content of cellulose, containing betulin with anticancer, antiviral (HIV-1), anti-inflammatory, and antiallergic properties (DARY PODLASIA company, (Bielsk Podlaski, Poland), Ogrodowa 87a/3) [birch bark (BB) —birch is characterized by rapid growth and resistance to harsh environmental conditions. Birch bark is distinguished by an exceptionally high content (approx. 80%) of triterpene compounds. The most important compounds belonging to this group include lupeol, betulinic acid, and betulin, which has the strongest biological activity. According to the latest scientific research, betulin and its derivatives have anticancer, antiviral (HIV-1), antiprotozoal, and anti-inflammatory properties. The use of preparations containing betulin can help people who suffer from skin diseases of allergic etiology (psoriasis, eczema). Betulin inhibits the secretion of histamine, thanks to which it has an anti-allergic effect. In addition, it has antioxidant properties, so it can be used in anti-aging prophylactic treatment of the skin. The antiviral effect of betulin also applies to the prevention of type 1 and 2 herpes simplex]. GUMOSIL B silicone rubber matrix polymer composites additionally contained magnetic particles with ferromagnetic properties in the form of carbonyl iron in the amount of 20% *w*/*w* obtained by decomposition of iron pentacarbonyl at high temperatures (97%, Alfa Aesar, Thermo Fisher Scientific, Oxford, UK), thanks to which it was possible to change the properties of composites in a constant magnetic field.

### 3.2. Preparation of Test Samples

All the test samples were prepared according to the same procedure. After the sample composition was developed, the individual components were weighed and added in the following order: GUMOSIL B silicone rubber, magnetic particles in the form of carbonyl iron (20% *w*/*w*), the filler (EG 290 expanded graphite, birch bark with betulin, 10% *w*/*w*) and the OL-1 hardener for silicone rubber. The whole was mixed using a mechanical stirrer at a speed of 300 rpm for 180 s. The mixed liquid mass of the sample was placed in molds prepared according to the PN-EN ISO 10210:2018-1 standard [18]. Before polymerization was started, the samples were divided into two groups. The first group of samples was placed between the poles of a laboratory electromagnet in a constant magnetic field with magnetic induction B ranging from 0 to 1 T, in which their polymerization was conducted. The second group of samples was placed outside a constant magnetic field (B = 0.0 T) during polymerization. The composition of the tested samples is given in Table 5.

The measurement of each of the polymer composite samples was repeated five times. The measurement points presented in the graphs are the mean values of these five measurements. Graphs 2, 3, 5, 7, 8, 9, and 10 have been corrected, introducing the error bars indicating the minimum and maximum values for each measurement point.

### 3.3. Testing Methodology

Mechanical tensile strength was tested using a Zwick/Roell Z050 type, KL 0.05 testing machine, with a 50 kN measuring head, a tensile speed of 50 mm/min, and a speed of 5 mm/min for the module (Zwick Roell, Ulm, Germany). The tensile strength was tested in accordance with the PN EN ISO 527-1 [19] and PN EN ISO 527-2 standards [20]. The surface wetting angle and the surface free energy were determined using a DSA 25E goniometer from KRUSS GmbH, Hamburg (Germany). Microscopic studies of polymer composite surfaces were carried out using the FEI Quanta 3D FEGSEM scanning electron microscope with an additional FIB ion column integrated with the EDAX Trident system. Analysis of the chemical composition in microareas was performed with X-ray energy dispersion spectroscopy (EDXS—Apollo 40 company EDAX) using the ZAF correction procedure. A constant magnetic field with magnetic induction B of 0.0–1.0 T was produced using an LS-EM-7V model laboratory electromagnet with an LS-648 control power supply, and magnetic induction was measured using an LS-F41-FC teslometer and an LS-FP-2X-250-TF15 Hall probe (all devices manufactured by Lake Shore Cryotronics, Westerville, OH, USA). The water absorbability of the samples was tested as follows. The samples were cut into smaller fragments, described, and weighed on an analytical balance. Then, the samples were placed in a glass crystallizer filled with deionized water for 24 h. After 24 h, the samples were removed from deionized water, dried at room temperature for 30 min, and weighed. The water absorbability of the sample *n*_w_ was calculated from the following Equation (1):

(1) $$n_{w}=\frac{m_{n}-m}{m}\;\cdot 100\%$$

where *m*_n_ is the mass (in g) of the sample saturated with distilled water and *m* is the mass (in g) of the dry sample. The frost resistance of the samples was tested as follows. The samples dried to constant weight were immersed completely in distilled water for 24 h. After their removal from distilled water, the samples were placed on tissue paper for 30 min to dry and then placed in the freezer at −20 °C for 24 h. After removal from the freezer, the samples were immersed completely in distilled water again for 24 h, then removed from the water, air-dried for 48 h, and weighed. The percentage damage of sample *S* was defined as the relative loss of its mass calculated from Equation (2):

(2) $$S=\frac{m_{1}-m_{2}}{m_{1}}\cdot 100\%$$

where *m*_1_ is the mass (g) of the dried test sample and *m*_2_ is the mass (g) of the sample dried at the end of the test. There are many different methods of testing frost resistance. The frost resistance tests presented in this paper were based on the PN-EN 206+A1:2016-12 [21] and PN-88/B-06250 [22] standards, which describes the so-called normal frost resistance test, based on the study of weight loss as a result of repeated freezing and thawing of samples. In our research, frost resistance was defined as the percentage of weight loss of sample S due to damage by water freezing in that sample, such as cracks and chips. Thus, the lower the weight loss of the sample, the lower its percentage of damage and the higher its frost resistance. All quantities for each sample were measured five times and the results presented in the figures are the arithmetic means of the five measurements with the standard deviation.

## 4. Conclusions

This paper presents the results of our research on new polymer composites based on silicone rubber matrix. The polymer composites contained admixtures in the amount of 10% *w*/*w* of expanded graphite (EG) or birch bark (BB). Some polymer composites were additionally modified with magnetic particles in the form of carbonyl iron (Fe) in the amount of 20% *w*/*w*. In the work, a constant magnetic field with magnetic induction B was used to modify the properties and structure of the composites. As a result of the research on the water absorbability of the composites, it was found that the polymer composites with the admixtures of birch bark and birch bark with carbonyl iron were characterized by the highest water absorbability of 29.4%. A 10-fold increase in the water absorbability of the composite was observed, and a 20-fold increase in the case of the composite obtained in a constant magnetic field. The best effect was obtained in a constant magnetic field (B = 0.5 T) for the composite with an admixture of EG 290 expanded graphite, where the water absorbability of the composite decreased from 0.4647% to 0.0875%, i.e., five times. The deterioration of frost resistance (weight loss) was the greatest for the composite containing an admixture of birch bark. Frost resistance deteriorated 18 times for a sample obtained without a magnetic field and 22 times for a sample obtained in a constant magnetic field with B = 0.5 T. An almost two-fold frost resistance improvement (the smallest loss of mass) occurred for a composite containing an admixture of carbonyl iron, especially under the influence of a constant magnetic field. In addition to the observation that the value of magnetic induction B affects the ultimate tensile strength and elongation of polymer composites, it was also demonstrated that the composites acquired the properties of anisotropic materials in a constant magnetic field. On examination of the stress values σ_m_ of the composites, it was found that they are much higher when the direction of tensile force **F** is perpendicular to the direction of magnetic induction B during the polymerization of the composite. The difference in the value of stress in the material, with the action of a constant magnetic field, e.g., B = 0.5 T, reached approx. 55%, while the difference in the deformation of the material was approx. 35%. The constant magnetic field increased the surface wetting angle of the carbonyl iron and birch bark polymer composite by 25.3%, and that of the composite with an admixture of carbonyl iron and expanded graphite by 19.6%. The wettability of the surface became lower, and thus the surface of the composites became more and more hydrophobic. On the other hand, the constant magnetic field reduced the surface free energy of the polymer composite with an admixture of carbonyl iron and birch bark by 34%, and that of the composite with an admixture of carbonyl iron and expanded graphite by 29.4%. The composites obtained in a constant magnetic field had visible, larger clusters of magnetic particles, which were arranged into chains. On the basis of SEM microscopic images, divided into microareas by the EDXS method, chemical composition analysis was performed. The dominant component in many microareas was silicon (Si) due to its contribution to silicone rubber. Some of the microareas demonstrated the presence of carbonyl iron particles arranged in chains. The quantitative content of carbon (C) was not taken into account in the studies, because this component comes from both expanded graphite and silicone rubber, so it would be overestimated.

A constant magnetic field, by affecting the multiphase structure of the polymer composite, changes the thermodynamic equilibrium of the material. Under the influence of a constant magnetic field, thermodynamic functions such as enthalpy and internal energy can change. Thanks to the use of magnetic particles, e.g., carbonyl iron, the shape of polymer composites can be controlled. They were distributed in the polymer and converted the energy of the magnetic field into heat. The appropriate temperature could be achieved by changing the density of the magnetic particles and the value of magnetic induction.

The only limitation in the use of a constant magnetic field to obtain samples of polymer composites may be the space between the pole pieces of an electromagnet producing a constant magnetic field with a specific magnetic induction B. This space determines the dimensions of the individual samples of polymer composites intended for testing.

## Figures and Tables

**Table 1:** The content of some elements in the GUM(B)OL-1(Fe)(−) composite, obtained without the use of a magnetic field (B = 0), in the marked microareas 10, 11, and 12 (Figure 13).

EDXS (% wt)			
Selected Fragment of the Composite	10	11	12
O	31.6	38.5	12.0
Na	0.9	0.3	0.3
Al	1.9	0.8	0.3
Si	53.9	57.6	14.2
Fe	11.8	2.8	73.2

**Table 2:** The content of some elements in the GUM(B)OL-1(Fe)(−) composite, obtained with the participation of a constant magnetic field (B = 0.25 T), in the marked microareas 1, 2, 3, and 4 (Figure 13).

EDXS (% wt)				
Selected Fragment of the Composite	1	2	3	4
O	32.4	18.0	40.7	38.7
Na	1.0	0.4	0.7	1.1
Al	1.8	0.3	1.7	3.8
Si	55.4	16.9	53.3	52.9
Fe	9.4	64.4	3.6	3.5

**Table 3:** The content of some elements in the GUM(B)OL-1(Fe)(EG) composite, obtained with the participation of a constant magnetic field (B = 0.25 T), in the marked microareas 1–8 (Figure 13).

EDXS (% *w/w*)								
Selected Fragment of the Composite	1	2	3	4	5	6	7	8
O	33.4	42.5	13.6	61.2	46.6	26.7	37.4	45.7
Na	0.7	0.6	-	1.1	0.5	-	-	20.4
Al	1.4	1.2	0.3	0.8	1.0	1.4	3.2	0.5
Si	52.5	52.6	16.7	23.5	48.4	60.1	51.8	14.2
S	0.9	-	-	8.8	1.1	0.4	1.6	14.4
Fe	11.1	3.2	69.4	4.6	2.4	11.4	6.0	2.4

**Table 4:** Parameters of polymer composites 4 [GUM(B)OL-1(Fe)(BB)] and 6 [GUM(B)OL-1(Fe)(EG)], obtained in a constant magnetic field with induction B = 0.5 T and without the participation of a magnetic field.

Number and Type	B (T)	n_w_ (%)	S (%)	σ_m_ (MPa)		Ɛ_m_ (%)		θ (Grade)	SFE (mJ·m^−2^)
				F||B	F⊥B	F||B	F⊥B		
4 GUM(B)OL-1(Fe)(BB)	0	2.51	3.09	-	-	-	-	86.6	41.5
	0.5	2.11	2.82	-	-	-	-	93.0	38.3
6 GUM(B)OL-1(Fe)(EG)	0	0.68	0.63	0.36	0.35	25	35	93.8	37.3
	0.5	0.49	0.68	0.31	0.48	20	27	104.0	31.0

**Table 5:** List of tested polymer composites on the basis of silicone rubber.

Number	Composite Marking	The Content of the Composite				
		Silicone Rubber (% *w/w*)	Carbonyl Iron (% *w/w*)	Birch Bark (% *w/w*)	Expanded Graphite EG 290 (% *w/w*)	Hardener for Silicone Rubber OL-1 (% *w/w*)
1	GUM(B)OL-1(−)(−)	97	-	-	-	3
2	GUM(B)OL-1(Fe)(−)	77.5	20	-	-	2.5
3	GUM(B)OL-1(−)(BB)	87.3	-	10	-	2.7
4	GUM(B)OL-1(Fe)(BB)	67.9	20	10	-	2.1
5	GUM(B)OL-1(−)(EG)	87.3	-	-	10	2.7
6	GUM(B)OL-1(Fe)(EG)	67.9	20	-	10	2.1

## Data Availability

Data are contained within the article.
